# Supplementary material for: Characterization of the Complete Mitogenomes of Four Dacinae Species (Diptera: Tephritidae) with Phylogenetic Analysis
Source: Animals (Basel). 2025 Nov 15;15(22):3301. doi: 10.3390/ani15223301 (PMC12649545; doi:10.3390/ani15223301)
Supplement: Supplementary file 1 [file animals-15-03301-s001.zip › animals-3960197-supplementary.docx]

Characterization of the Complete Mitogenomes of Four Dacinae Species (Diptera: Tephritidae) With Phylogenetic Analysis

**Supplementary data**

**Table S1.** Mitogenomic organization of *Acroceratitis separata*, *Acrotaeniostola quadrivittata*, *Gastrozona parviseta*, and *Paragastrozona vulgaris*.

**Table S2.** Nucleotide composition of the mitogenome of *Acroceratitis separata.*

**Table S3.** Nucleotide composition of the mitogenome of *Acrotaeniostola quadrivittata.*

**Table S4.** Nucleotide composition of the mitogenome of *Gastrozona parviseta.*

**Table S5.** Nucleotide composition of the mitogenome of *Paragastrozona vulgaris.*

**Table S6.** The optimal partitioning schemes and nucleotide substitution models for BI and ML selected by PartitionFinder.

**Figure S1.** Hierarchical clustering analysis for the heatmap showing AA usage count of 46 mitogenomes species within Dacinae.

**Figure S2.** Hierarchical clustering analysis for the heatmap showing total codon counts of 46 mitogenomes species within Dacinae.

**Figure S3.** Predicted cloverleaf secondary structure for the 22 tRNAs of *Acroceratitis separata*.

**Figure S4.** Predicted cloverleaf secondary structure for the 22 tRNAs of *Acrotaeniostola quadrivittata*.

**Figure S5.** Predicted cloverleaf secondary structure for the 22 tRNAs of *Gastrozona parviseta*.

**Figure S6.** Predicted cloverleaf secondary structure for the 22 tRNAs of *Paragastrozona vulgaris*.

**Figure S7.** Phylogenetic tree of Dacinae inferred from the PCG123-BI + ML, PCG12R-BI + ML, and AA-ML datasets using Bayesian inference and Maximum likelihood methods. Numbers on branches are posterior probabilities and bootstrap support values.

**Figure S8.** Phylogenetic tree of Dacinae inferred from the PCG12-ML + BI and AA-BI datasets using Maximum likelihood and Bayesian inference methods. Numbers on branches are bootstrap support and posterior probabilities values.

**Figure S9.** Phylogeny of Dacinae inferred from the PCG123 and PCG123R datasets using PhyloBayes analysis under the site-heterogeneous mixture model CAT + GTR. Supports at nodes are Bayesian posterior probabilities.

**Figure S10.** Phylogeny of Dacinae inferred from the PCG12R dataset using PhyloBayes analysis under the site-heterogeneous mixture model CAT + GTR. Supports at nodes are Bayesian posterior probabilities.

**Figure S11.** Phylogeny of Dacinae inferred from the PCG12 dataset using PhyloBayes analysis under the site-heterogeneous mixture model CAT + GTR. Supports at nodes are Bayesian posterior probabilities.

**Table S1.** Mitogenomic organization of *Acroceratitis separata*, *Acrotaeniostola quadrivittata*, *Gastrozona parviseta*, and *Paragastrozona vulgaris.*

| Gene | Location | | Size (bp) | Intergenic  Nucleotides (bp) | Codon | | Strand |
| --- | --- | --- | --- | --- | --- | --- | --- |
|  | from | to |  |  | start | stop |  |
| *trnI* | 1/1/1/1 | 66/65/66/68 | 66/65/66/68 | 0/0/0/0 |  |  | H |
| *trnQ* | 64/70/77/70 | 132/138/145/138 | 69/69/69/69 | -3/4/10/1 |  |  | L |
| *trnM* | 141/141/145/145 | 209/209/213/213 | 69/69/69/69 | 8/2/-1/6 |  |  | H |
| *nad2* | 234/234/238/238 | 1241/1241/1236/1236 | 1008/1008/999/999 | 24/24/24/24 | ATA/ATG/ATA/ATA | TAG/TAG/TAG/TAG | H |
| *trnW* | 1244/1245/1240/1240 | 1311/1312/1307/1307 | 68/68/68/68 | 2/3/3/3 |  |  | H |
| *trnC* | 1304/1305/1300/1300 | 1367/1367/1363/1363 | 64/63/64/64 | -8/-8/-8/-8 |  |  | L |
| *trnY* | 1369/1369/1365/1365 | 1435/1434/1431/1431 | 67/66/67/67 | 1/1/1/1 |  |  | L |
| *cox1* | 1440/1439/1436/1436 | 2967/2966/2963/2963 | 1528/1528/1528/1528 | 4/4/4/4 | CAA/CAA/CAA/CAA | T/T/T/T | H |
| *trnL2* | 2969/2968/2965/2965 | 3034/3033/3030/3030 | 66/66/66/66 | 1/1/1/1 |  |  | H |
| *cox2* | 3039/3044/3035/3035 | 3728/3733/3724/3724 | 690/690/690/690 | 4/10/4/4 | ATG/ATG/ATG/ATG | TAA/TAA/TAA/TAA | H |
| *trnK* | 3734/3738/3730/3730 | 3804/3808/3800/3800 | 71/71/71/71 | 5/4/5/5 |  |  | H |
| *trnD* | 3806/3812/3802/3804 | 3872/3878/3868/3871 | 67/67/67/68 | 1/3/1/3 |  |  | H |
| *atp8* | 3873/3879/3869/3872 | 4034/4040/4030/4033 | 162/162/162/162 | 0/0/0/0 | ATT/ATA/ATT/ATT | TAA/TAA/TAA/TAA | H |
| *atp6* | 4028/4034/4024/4027 | 4705/4711/4701/4704 | 678/678/678/678 | -7/-7/-7/-7 | ATG/ATG/ATG/ATG | TAA/TAA/TAA/TAA | H |
| *cox3* | 4705/4711/4701/4704 | 5493/5499/5489/5492 | 789/789/789/789 | -1/-1/-1/-1 | ATG/ATG/ATG/ATG | TAA/TAA/TAA/TAA | H |
| *trnG* | 5503/5502/5496/5499 | 5567/5566/5560/5563 | 65/65/65/65 | 9/2/6/6 |  |  | H |
| *nad3* | 5568/5567/5561/5564 | 5921/5920/5914/5917 | 354/354/354/354 | 0/0/0/0 | ATT/ATT/ATC/ATC | TAA/TAA/TAA/TAA | H |
| *trnA* | 5930/5934/5921/5926 | 5994/5999/5984/5989 | 65/66/64/64 | 8/13/6/8 |  |  | H |
| *trnR* | 6086/6002/6089/6080 | 6149/6067/6152/6146 | 64/66/64/67 | 91/2/104/90 |  |  | H |
| *trnN* | 6154/6166/6159/6153 | 6219/6233/6223/6217 | 66/68/65/65 | 4/98/6/6 |  |  | H |
| *trnS1* | 6220/6234/6224/6218 | 6287/6301/6291/6285 | 68/68/68/68 | 0/0/0/0 |  |  | H |
| *trnE* | 6288/6302/6292/6286 | 6355/6368/6359/6353 | 68/67/68/68 | 0/0/0/0 |  |  | H |
| *trnF* | 6374/6387/6378/6372 | 6439/6454/6443/6437 | 66/68/66/66 | 18/18/18/18 |  |  | L |
| *nad5* | 6440/6455/6444/6438 | 8177/8189/8181/8175 | 1738/1735/1738/1738 | 0/0/0/0 | GTG/GTG/ATG/GTG | T/T/T/T | L |
| *trnH* | 8178/8190/8182/8176 | 8242/8253/8246/8240 | 65/64/65/65 | 0/0/0/0 |  |  | L |
| *nad4* | 8247/8255/8248/8242 | 9587/9595/9588/9582 | 1341/1341/1341/1341 | 4/1/1/1 | ATG/ATG/ATG/ATG | TAA/TAA/TAA/TAA | L |
| *nad4L* | 9581/9589/9582/9576 | 9877/9885/9878/9872 | 297/297/297/297 | -7/-7/-7/-7 | ATG/ATG/ATG/ATG | TAA/TAA/TAA/TAA | L |
| *trnT* | 9880/9888/9881/9875 | 9944/9954/9947/9939 | 65/67/67/65 | 2/2/2/2 |  |  | H |
| *trnP* | 9945/9955/9948/9940 | 10010/10021/10013/10005 | 66/67/66/66 | 0/0/0/0 |  |  | L |
| *nad6* | 10016/10027/10019/10011 | 10537/10545/10540/10532 | 522/519/522/522 | 5/5/5/5 | ATA/ATA/ATA/ATA | TAA/TAA/TAA/TAA | H |
| *cytb* | 10537/10545/10540/10532 | 11673/11681/11676/11668 | 1137/1137/1137/1137 | -1/-1/-1/-1 | ATG/ATG/ATG/ATG | TAA/TAG/TAG/TAG | H |
| *trnS2* | 11680/11680/11675/11667 | 11747/11746/11741/11733 | 68/67/67/67 | 6/-2/-2/-2 |  |  | H |
| *nad1* | 11754/11753/11750/11742 | 12693/12692/12688/12680 | 940/940/939/939 | 6/6/8/8 | ATT/ATT/ATT/ATT | T/T/TAA/TAA | L |
| *trnL1* | 12713/12712/12708/12700 | 12777/12776/12772/12764 | 65/65/65/65 | 19/19/19/19 |  |  | L |
| *rrnL* | 12778/12777/12773/12765 | 14111/14115/14105/14098 | 1334/1339/1333/1334 | 0/0/0/0 |  |  | L |
| *trnV* | 14112/14116/14106/14099 | 14183/14187/14177/14170 | 72/72/72/72 | 0/0/0/0 |  |  | L |
| *rrnS* | 14184/14188/14178/14171 | 14980/14980/14974/14958 | 797/793/797/788 | 0/0/0/0 |  |  | L |
| *CR* | 14981/14981/14975/14959 | 16603/16112/16691/16594 | 1623/1132/1717/1636 | 0/0/0/0 |  |  | H |

**Table S2.** Nucleotide composition of the mitogenome of *Acroceratitis separata.*

| Regions | Size (bp) | T(U) % | C % | A % | G % | AT % | GC % | GT % | AT skew | GC skew |
| --- | --- | --- | --- | --- | --- | --- | --- | --- | --- | --- |
| PCGs | 11181 | 44.3 | 11.5 | 31.8 | 12.4 | 76.1 | 23.9 | 56.7 | -0.163 | 0.04 |
| 1st codon position | 3727 | 37.8 | 11.1 | 30.9 | 20.2 | 68.7 | 31.3 | 58 | -0.1 | 0.29 |
| 2nd codon position | 3727 | 45.5 | 19.4 | 20.5 | 14.5 | 66 | 33.9 | 60 | -0.378 | -0.144 |
| 3rd codon position | 3727 | 49.5 | 3.9 | 44.1 | 2.5 | 93.6 | 6.4 | 52 | -0.058 | -0.205 |
| A+T-rich region | 1623 | 50 | 8.7 | 32.1 | 9.1 | 82.1 | 17.8 | 59.1 | -0.218 | 0.021 |
| *atp6* | 678 | 40.9 | 16.1 | 32.2 | 10.9 | 73.1 | 27 | 51.8 | -0.119 | -0.191 |
| *atp8* | 162 | 42.6 | 13.6 | 37 | 6.8 | 79.6 | 20.4 | 49.4 | -0.07 | -0.333 |
| *cox1* | 1528 | 40.4 | 14.4 | 30.4 | 14.7 | 70.8 | 29.1 | 55.1 | -0.141 | 0.011 |
| *cox2* | 690 | 37.8 | 14.6 | 35.4 | 12.2 | 73.2 | 26.8 | 50 | -0.034 | -0.092 |
| *cox3* | 789 | 39.5 | 15.6 | 30.8 | 14.1 | 70.3 | 29.7 | 53.6 | -0.124 | -0.051 |
| *cytb* | 1137 | 41.3 | 14 | 32.4 | 12.3 | 73.7 | 26.3 | 53.6 | -0.122 | -0.064 |
| *nad1* | 940 | 49.6 | 8.1 | 28.3 | 14 | 77.9 | 22.1 | 63.6 | -0.273 | 0.269 |
| *nad2* | 1008 | 44.8 | 12.2 | 34.9 | 8 | 79.7 | 20.2 | 52.8 | -0.124 | -0.206 |
| *nad3* | 354 | 46.9 | 11.9 | 32.8 | 8.5 | 79.7 | 20.4 | 55.4 | -0.177 | -0.167 |
| *nad4* | 1341 | 48.5 | 7.6 | 29.9 | 13.9 | 78.4 | 21.5 | 62.4 | -0.238 | 0.294 |
| *nad4L* | 297 | 51.5 | 5.7 | 30.3 | 12.5 | 81.8 | 18.2 | 64 | -0.259 | 0.37 |
| *nad5* | 1738 | 47.9 | 7.8 | 30.3 | 14 | 78.2 | 21.8 | 61.9 | -0.226 | 0.288 |
| *nad6* | 522 | 43.1 | 10.2 | 40.4 | 6.3 | 83.5 | 16.5 | 49.4 | -0.032 | -0.233 |
| *rrnL* | 1334 | 43.8 | 6.5 | 37.3 | 12.4 | 81.1 | 18.9 | 56.2 | -0.079 | 0.31 |
| *rrnS* | 797 | 41.4 | 8.3 | 36.6 | 13.7 | 78 | 22 | 55.1 | -0.061 | 0.246 |
| *rRNAs* | 2131 | 42.9 | 7.2 | 37.1 | 12.9 | 80 | 20.1 | 55.8 | -0.073 | 0.283 |
| *tRNAs* | 1470 | 38.8 | 9.8 | 38.4 | 12.9 | 77.2 | 22.7 | 51.7 | -0.005 | 0.138 |
| Full genome | 16603 | 38.4 | 13.1 | 39 | 9.5 | 77.4 | 22.6 | 47.9 | 0.008 | -0.157 |

**Table S3.** Nucleotide composition of the mitogenome of *Acrotaeniostola quadrivittata.*

| Regions | Size (bp) | T(U) % | C % | A % | G % | AT % | GC % | GT % | AT skew | GC skew |
| --- | --- | --- | --- | --- | --- | --- | --- | --- | --- | --- |
| PCGs | 11175 | 44.8 | 11 | 32.6 | 11.5 | 77.4 | 22.5 | 56.3 | -0.158 | 0.022 |
| 1st codon position | 3725 | 37.8 | 11.1 | 32.4 | 18.7 | 70.2 | 29.8 | 56.5 | -0.077 | 0.255 |
| 2nd codon position | 3725 | 46.1 | 19 | 20.8 | 14.1 | 66.9 | 33.1 | 60.2 | -0.378 | -0.148 |
| 3rd codon position | 3725 | 50.6 | 3 | 44.5 | 1.9 | 95.1 | 4.9 | 52.5 | -0.064 | -0.238 |
| A+T-rich region | 1132 | 40.4 | 10.6 | 39.4 | 9.6 | 79.8 | 20.2 | 50 | -0.012 | -0.048 |
| *atp6* | 678 | 42.6 | 14.2 | 33.3 | 9.9 | 75.9 | 24.1 | 52.5 | -0.122 | -0.178 |
| *atp8* | 162 | 41.4 | 13 | 40.1 | 5.6 | 81.5 | 18.6 | 47 | -0.015 | -0.4 |
| *cox1* | 1528 | 39.8 | 14.1 | 31.5 | 14.6 | 71.3 | 28.7 | 54.4 | -0.117 | 0.016 |
| *cox2* | 690 | 39 | 14.5 | 35.2 | 11.3 | 74.2 | 25.8 | 50.3 | -0.051 | -0.124 |
| *cox3* | 789 | 41.3 | 14.1 | 31.3 | 13.3 | 72.6 | 27.4 | 54.6 | -0.138 | -0.028 |
| *cytb* | 1137 | 42.1 | 13.4 | 32.9 | 11.6 | 75 | 25 | 53.7 | -0.123 | -0.07 |
| *nad1* | 940 | 51.3 | 8.1 | 27.8 | 12.9 | 79.1 | 21 | 64.2 | -0.297 | 0.228 |
| *nad2* | 1008 | 45.8 | 11.5 | 35 | 7.6 | 80.8 | 19.1 | 53.4 | -0.134 | -0.202 |
| *nad3* | 354 | 44.6 | 12.7 | 35 | 7.6 | 79.6 | 20.3 | 52.2 | -0.121 | -0.25 |
| *nad4* | 1341 | 48.8 | 7.6 | 31.5 | 12.2 | 80.3 | 19.8 | 61 | -0.216 | 0.23 |
| *nad4L* | 297 | 53.9 | 5.7 | 29 | 11.4 | 82.9 | 17.1 | 65.3 | -0.301 | 0.333 |
| *nad5* | 1735 | 48.2 | 7.4 | 31.4 | 13 | 79.6 | 20.4 | 61.2 | -0.211 | 0.271 |
| *nad6* | 519 | 43 | 10.2 | 41.2 | 5.6 | 84.2 | 15.8 | 48.6 | -0.021 | -0.293 |
| *rrnL* | 1339 | 43.5 | 6.1 | 39.3 | 11.1 | 82.8 | 17.2 | 54.6 | -0.051 | 0.287 |
| *rrnS* | 793 | 41 | 7.4 | 38.8 | 12.7 | 79.8 | 20.1 | 53.7 | -0.027 | 0.263 |
| *rRNAs* | 2132 | 42.6 | 6.6 | 39.1 | 11.7 | 81.7 | 18.3 | 54.3 | -0.042 | 0.277 |
| *tRNAs* | 1474 | 40.1 | 9.1 | 38.6 | 12.2 | 78.7 | 21.3 | 52.3 | -0.019 | 0.146 |
| Full genome | 16112 | 38.1 | 12.4 | 40.3 | 9.1 | 78.4 | 21.5 | 47.2 | 0.027 | -0.153 |

**Table S4.** Nucleotide composition of the mitogenome of *Gastrozona parviseta.*

| Regions | Size (bp) | T(U) % | C % | A % | G % | AT % | GC % | GT % | AT skew | GC skew |
| --- | --- | --- | --- | --- | --- | --- | --- | --- | --- | --- |
| PCGs | 11172 | 42.8 | 13.3 | 30.8 | 13.1 | 73.6 | 26.4 | 55.9 | -0.163 | -0.007 |
| 1st codon position | 3724 | 36.6 | 12.6 | 30.4 | 20.4 | 67 | 33 | 57 | -0.093 | 0.238 |
| 2nd codon position | 3724 | 45.4 | 19.9 | 20.4 | 14.4 | 65.8 | 34.3 | 59.8 | -0.38 | -0.162 |
| 3rd codon position | 3724 | 46.3 | 7.5 | 41.6 | 4.6 | 87.9 | 12.1 | 50.9 | -0.054 | -0.237 |
| A+T-rich region | 1717 | 45 | 11.5 | 34.3 | 9.2 | 79.3 | 20.7 | 54.2 | -0.135 | -0.11 |
| *atp6* | 678 | 41 | 17.6 | 30.5 | 10.9 | 71.5 | 28.5 | 51.9 | -0.146 | -0.233 |
| *atp8* | 162 | 40.1 | 16 | 35.8 | 8 | 75.9 | 24 | 48.1 | -0.057 | -0.333 |
| *cox1* | 1528 | 38.2 | 16.8 | 30 | 15.1 | 68.2 | 31.9 | 53.3 | -0.121 | -0.053 |
| *cox2* | 690 | 36.5 | 17.5 | 33.3 | 12.6 | 69.8 | 30.1 | 49.1 | -0.046 | -0.163 |
| *cox3* | 789 | 37 | 18.9 | 30.2 | 13.9 | 67.2 | 32.8 | 50.9 | -0.102 | -0.151 |
| *cytb* | 1137 | 37.6 | 17.9 | 31.8 | 12.8 | 69.4 | 30.7 | 50.4 | -0.085 | -0.167 |
| *nad1* | 939 | 50.2 | 8.2 | 25.3 | 16.3 | 75.5 | 24.5 | 66.5 | -0.329 | 0.33 |
| *nad2* | 999 | 43.1 | 13.9 | 34 | 8.9 | 77.1 | 22.8 | 52 | -0.118 | -0.219 |
| *nad3* | 354 | 44.6 | 14.7 | 32.2 | 8.5 | 76.8 | 23.2 | 53.1 | -0.162 | -0.268 |
| *nad4* | 1341 | 48.2 | 7.8 | 29.2 | 14.8 | 77.4 | 22.6 | 63 | -0.245 | 0.314 |
| *nad4L* | 297 | 50.8 | 6.7 | 28.3 | 14.1 | 79.1 | 20.8 | 64.9 | -0.285 | 0.355 |
| *nad5* | 1738 | 46.4 | 8.4 | 29.9 | 15.2 | 76.3 | 23.6 | 61.6 | -0.216 | 0.29 |
| *nad6* | 522 | 41.6 | 14.8 | 37.7 | 5.9 | 79.3 | 20.7 | 47.5 | -0.048 | -0.426 |
| *rrnL* | 1333 | 42.7 | 6.9 | 37.6 | 12.8 | 80.3 | 19.7 | 55.5 | -0.064 | 0.3 |
| *rrnS* | 797 | 41.4 | 8.5 | 35.5 | 14.6 | 76.9 | 23.1 | 56 | -0.077 | 0.261 |
| *rRNAs* | 2130 | 42.2 | 7.5 | 36.8 | 13.5 | 79 | 21 | 55.7 | -0.068 | 0.284 |
| *tRNAs* | 1469 | 38.7 | 10.6 | 37.4 | 13.3 | 76.1 | 23.9 | 52 | -0.016 | 0.117 |
| Full genome | 16691 | 36.7 | 15 | 38.4 | 9.9 | 75.1 | 24.9 | 46.6 | 0.023 | -0.204 |

**Table S5.** Nucleotide composition of the mitogenome of *Paragastrozona vulgaris.*

| Regions | Size (bp) | T(U) % | C % | A % | G % | AT % | GC % | GT % | AT skew | GC skew |
| --- | --- | --- | --- | --- | --- | --- | --- | --- | --- | --- |
| PCGs | 11172 | 43.4 | 13 | 30.8 | 12.8 | 74.2 | 25.8 | 56.2 | -0.17 | -0.008 |
| 1st codon position | 3724 | 36.5 | 12.7 | 30.3 | 20.5 | 66.8 | 33.2 | 57 | -0.094 | 0.234 |
| 2nd codon position | 3724 | 45.4 | 19.7 | 20.4 | 14.4 | 65.8 | 34.1 | 59.8 | -0.379 | -0.156 |
| 3rd codon position | 3724 | 48.2 | 6.6 | 41.6 | 3.5 | 89.8 | 10.1 | 51.7 | -0.073 | -0.305 |
| A+T-rich region | 1636 | 34.3 | 12.5 | 40.4 | 12.8 | 74.7 | 25.3 | 47.1 | 0.082 | 0.014 |
| *atp6* | 678 | 41.3 | 16.8 | 30.8 | 11.1 | 72.1 | 27.9 | 52.4 | -0.145 | -0.206 |
| *atp8* | 162 | 42 | 13.6 | 37.7 | 6.8 | 79.7 | 20.4 | 48.8 | -0.054 | -0.333 |
| *cox1* | 1528 | 38.5 | 17 | 29.9 | 14.7 | 68.4 | 31.7 | 53.2 | -0.125 | -0.072 |
| *cox2* | 690 | 38 | 16.2 | 33.2 | 12.6 | 71.2 | 28.8 | 50.6 | -0.067 | -0.126 |
| *cox3* | 789 | 38.9 | 18.1 | 28.9 | 14.1 | 67.8 | 32.2 | 53 | -0.148 | -0.126 |
| *cytb* | 1137 | 37.6 | 17.3 | 32.7 | 12.3 | 70.3 | 29.6 | 49.9 | -0.07 | -0.169 |
| *nad1* | 939 | 50.7 | 8.5 | 24.9 | 15.9 | 75.6 | 24.4 | 66.6 | -0.341 | 0.301 |
| *nad2* | 999 | 42.8 | 15 | 33.4 | 8.7 | 76.2 | 23.7 | 51.5 | -0.123 | -0.266 |
| *nad3* | 354 | 44.6 | 14.1 | 33.1 | 8.2 | 77.7 | 22.3 | 52.8 | -0.149 | -0.266 |
| *nad4* | 1341 | 48.6 | 7.5 | 29.5 | 14.4 | 78.1 | 21.9 | 63 | -0.244 | 0.317 |
| *nad4L* | 297 | 51.2 | 6.7 | 28.3 | 13.8 | 79.5 | 20.5 | 65 | -0.288 | 0.344 |
| *nad5* | 1738 | 47.7 | 7.9 | 29.7 | 14.7 | 77.4 | 22.6 | 62.4 | -0.232 | 0.301 |
| *nad6* | 522 | 42.3 | 13.6 | 38.5 | 5.6 | 80.8 | 19.2 | 47.9 | -0.047 | -0.42 |
| *rrnL* | 1334 | 43.5 | 6.7 | 37.3 | 12.5 | 80.8 | 19.2 | 56 | -0.076 | 0.305 |
| *rrnS* | 788 | 40.9 | 8.5 | 35.8 | 14.8 | 76.7 | 23.3 | 55.7 | -0.066 | 0.272 |
| *rRNAs* | 2122 | 42.5 | 7.4 | 36.8 | 13.4 | 79.3 | 20.8 | 55.9 | -0.073 | 0.291 |
| *tRNAs* | 1473 | 38.3 | 10.7 | 37.7 | 13.3 | 76 | 24 | 51.6 | -0.008 | 0.107 |
| Full genome | 16594 | 35.7 | 14.8 | 39.4 | 10 | 75.1 | 24.8 | 45.7 | 0.049 | -0.193 |

**Table S6.** The optimal partitioning schemes and nucleotide substitution models for BI and ML selected by PartitionFinder.

| Data matrix | Subset partitions | Model |
| --- | --- | --- |
| PCG123-BI | P1: *cox1*_codon1, *cytb*_codon1, *atp6*_odon1, *cox3*_codon1, *cox2*_codon1 | GTR+I+G |
|  | P2: *cox3*_codon2, *atp6*_codon2, *cox1*_codon2, *cox2*_codon2, *cytb*_codon2 | GTR+I+G |
|  | P3: *cox3*_codon3, *cytb*_codon3, *atp6*_codon3 | HKY+I+G |
|  | P4: *nad3*_codon1, *nad2*_codon1, *nad6*_codon1, *atp8*_codon1 | GTR+I+G |
|  | P5: *atp8*_codon2, *nad3*_codon2, *nad2*_codon2, *nad6*_codon2 | GTR+I+G |
|  | P6: *atp8*_codon3, *cox2*_codon3, *nad3*_codon3, *nad6*_codon3 | HKY+I+G |
|  | P7: *cox1*_codon3, *nad2*_codon3 | GTR+I+G |
|  | P8: *nad4L*_codon1, *nad1*_codon1, *nad5*_codon1, *nad4*_codon1 | GTR+I+G |
|  | P9: *nad4L*_codon2, *nad5*_codon2, *nad1*_codon2, *nad4*_codon2 | GTR+I+G |
|  | P10: *nad5*_codon3, *nad4*_codon3, *nad4L*_codon3, *nad1*_codon3 | GTR+I+G |
| PCG123-ML | P1: *cytb*_codon1, *cox1*_codon1, *atp6*_codon1, *cox3*_codon1, *cox2*_codon1 | GTR+I+G |
|  | P2: *cox3*_codon2, *atp6*_codon2, *cox2*_codon2, *cytb*_codon2, *cox1*_codon2 | TVM+I+G |
|  | P3: *cytb*_codon3, *atp6*_codon3, *cox3*_codon3, *cox2*_codon3 | TRN+I+G |
|  | P4: *nad3*_codon1, *nad2*_codon1, *nad6*_codon1, *atp8*_codon1 | GTR+I+G |
|  | P5: *atp8*_codon2, *nad3*_codon2, *nad6*_codon2, *nad2*_codon2 | TVM+I+G |
|  | P6: *atp8*_codon3, *nad3*_codon3, *nad6*_codon3 | TRN+G |
|  | P7: *cox1*_codon3 | HKY+I+G |
|  | P8: *nad4L*_codon1, *nad1*_codon1, *nad5*_codon1, *nad4*_codon1 | GTR+I+G |
|  | P9: *nad5*_codon2, *nad4L*_codon2, *nad1*_codon2, *nad4*_codon2 | GTR+I+G |
|  | P10: *nad5*_codon3, *nad1*_codon3, *nad4L*_codon3, *nad4*_codon3 | GTR+I+G |
|  | P11: *nad2*_codon3 | TRN+G |
| PCG123R-BI | P1: *cox1*_codon1, *cytb*_codon1, *atp6*_codon1, *cox3*_codon1, *cox2*_codon1 | GTR+I+G |
|  | P2: *cox3*_codon2, *atp6*_codon2, *cox1*_codon2, *cytb*_codon2, *cox2*_codon2 | GTR+I+G |
|  | P3: *cox3*_ codon3, *cytb*_codon3, *atp6*_codon3 | HKY+I+G |
|  | P4: *nad3*_codon1, *nad2*_ codon1, *nad6*_codon1, *atp8*_codon1 | GTR+I+G |
|  | P5: *atp8*_ codon2, *nad3*_codon2, *nad6*_codon2, *nad2*_codon2 | GTR+I+G |
|  | P6: *cox2*_codon3, *atp8*_codon3, *nad3*_codon3, *nad6*_codon3 | HKY+I+G |
|  | P7: *nad2*_codon3, *cox1*_codon3 | GTR+I+G |
|  | P8: *nad4L*_codon1, *nad1*_codon1, *nad5*_codon1, *nad4*_codon1 | GTR+I+G |
|  | P9: *nad4L*_codon2, *nad5*_codon2, *nad1*_codon2, *nad4*_codon2 | GTR+I+G |
|  | P10: *nad5*_codon3, *nad4*_codon3, *nad4L*_codon3, *nad1*_codon3 | GTR+I+G |
|  | P11: *rrnL*, *rrnS* | GTR+G |
| PCG123R-ML | P1: *cox1*_codon1, *cytb*_codon1, *atp6*_codon1, *cox2*_codon1, *cox3*_codon1 | GTR+I+G |
|  | P2: *cox3*_codon2, *atp6*_codon2, *cox2*_codon2, *cytb*_codon2, *cox1*_codon2 | TVM+I+G |
|  | P3: *cox1*_codon3, *cytb*_codon3, *atp6*_codon3, *cox3*_codon3, *cox2*_codon3 | GTR+I+G |
|  | P4: *nad3*_codon1, *nad2*_codon1, *nad6*_codon1, *atp8*_codon1 | GTR+I+G |
|  | P5: *atp8*_codon2, *nad3*_codon2, *nad6*_codon2, *nad2*_codon2 | TVM+I+G |
|  | P6: *atp8*_codon3, *nad3*_codon3, *nad6*_codon3 | TRN+G |
|  | P7: *nad4L*_codon1, *nad1*_codon1, *nad5*_codon1, *nad4*_codon1 | GTR+I+G |
|  | P8: *nad5*_codon2, *nad4L*_codon2, *nad1*_codon2, *nad4*_codon2 | GTR+I+G |
|  | P9: *nad5*_codon3, *nad1*_codon3, *nad4L*_codon3, *nad4*_codon3 | GTR+I+G |
|  | P10: *nad2*_codon3 | TRN+G |
|  | P11: *rrnS*, *rrnL* | GTR+G |
| PCG12-BI | P1: *cytb*_codonA, *cox1*_codonA, *atp6*_codonA, *cox3*_codonA, *cox2*_codonA | GTR+I+G |
|  | P2: *cox3*_codonB, *atp6*_codonB, *cox1*_codonB, *cytb*_codonB, *cox2*_codonB | GTR+I+G |
|  | P3: *nad2*_codonA, *nad3*_codonA, *nad6*_codonA, *atp8*_codonA | GTR+I+G |
|  | P4: *atp8*_codonB, *nad3*_codonB, *nad6*_codonB, *nad2*_codonB | GTR+I+G |
|  | P5: *nad4L*_codonA, *nad1*_codonA, *nad5*_codonA, *nad4*_codonA | GTR+I+G |
|  | P6: *nad4L*_codonB, *nad5*_codonB, *nad1*_codonB, *nad4*_codonB | GTR+I+G |
| PCG12-ML | P1: *cytb*_codonA, *cox1*_codonA, *atp6*_codonA, *cox2*_codonA, *cox3*_codonA | GTR+I+G |
|  | P2: *cox3*_codonB, *atp6*_codonB, *cox2*_codonB, *cytb*_codonB, *cox1*_codonB | TVM+I+G |
|  | P3: *nad6*_codonA, *atp8*_codonA | GTR+I+G |
|  | P4: *atp8*_codonB, *nad3*_codonB, *nad2*_codonB, *nad6*_codonB | TVM+I+G |
|  | P5: *nad5*_codonA, *nad4*_codonA, *nad1*_codonA, *nad4L*_codonA | GTR+I+G |
|  | P6: *nad5*_codonB, *nad4L*_codonB, *nad1*_codonB, *nad4*_codonB  P7: *nad2*_codonA, *nad3*_codonA | GTR+I+G  TRN+I+G |
| PCG12R-BI | P1: *cytb*_codonA, *cox1*_codonA, *cox3*_codonA, *atp6*_codonA, *cox2*_codonA  P2: *cox3*_codonB, *atp6*_codonB, *cox1*_codonB, *cytb*_codonB, *cox2*_codonB  P3: *nad2*_codonA, *nad3*_codonA, *nad6*_codonA, *atp8*_codonA  P4: *atp8*_codonB, *nad3*_codonB, *nad6*_codonB, *nad2*_codonB  P5: *nad4L*_codonA, *nad1*_codonA, *nad4*_codonA, *nad5*_codonA  P6: *nad5*_codonB, *nad4L*_codonB, *nad1*_codonB, *nad4*_codonB  P7: *rrnL*, *rrnS* | \| GTR+I+G \| \| --- \| \| GTR+I+G \| \| GTR+I+G \| \| GTR+I+G \| \| GTR+I+G \| \| GTR+I+G \| \| GTR+G \| |
| PCG12R-ML | P1: *cytb*_codonA, *cox1*_codonA, *cox3*_codonA, *cox2*_codonA, *atp6*_codonA  P2: *cox3*_codonB, *atp6*_codonB, *cox2*_codonB, *cytb*_codonB, *cox1*_codonB  P3: *nad6*_codonA, *atp8*_codonA  P4: *atp8*_codonB, *nad3*_codonB, *nad2*_codonB, *nad6*_codonB  P5: *nad5*_codonA, *nad1*_codonA, *nad4*_codonA, *nad4L*_codonA  P6: *nad5*_codonB, *nad4L*_codonB, *nad1*_codonB, *nad4*_codonB  P7: *nad2*_codonA, *nad3*_codonA  P8: *rrnL*, *rrnS* | \| GTR+I+G \| \| --- \| \| TVM+I+G \| \| GTR+I+G \| \| TVM+I+G \| \| TIM+I+G \| \| GTR+I+G \| \| TRN+I+G  GTR+G \| |
| AA-BI | P1: *nad4L*, *cox2*, *atp6*, *nad1*  P2: *nad2*, *atp8*  P3: *cox1*  P4: *cox3*, *cytb*  P5: *nad3*, *nad4*  P6: *nad6*, *nad5* | MTMAM+I+G |
|  |  | MTMAM+G |
|  |  | MTMAM+I+G |
|  |  | MTMAM+I+G  MTMAM+I+G  MTMAM+I+G |
| AA-ML | P1: *atp6*, *cox2* | MTMAM+I+G  MTMAM+I+G+F  MTMAM+I+G  MTMAM+I+G  MTART+I+G+F  MTART+G |
|  | P2: *atp8*, *nad3*, *nad6*, *nad2* |  |
|  | P3: *cox1*  P4: *cytb*, *cox3*  P5: *nad4L*, *nad5*, *nad1*  P6: *nad4* |  |


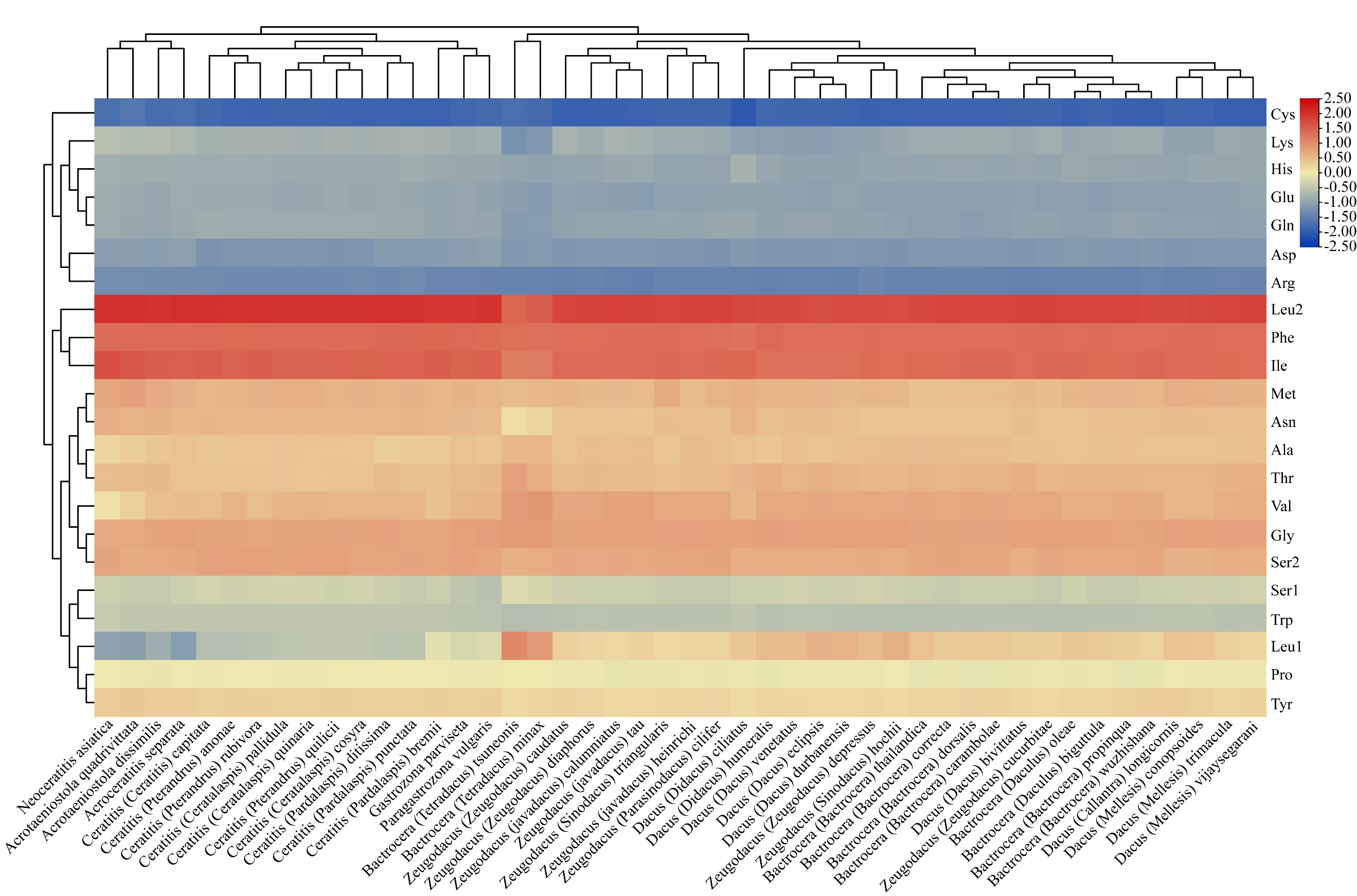


**Figure S1.** Hierarchical clustering analysis for the heatmap showing AA usage count of 46 mitogenomes species within Dacinae.


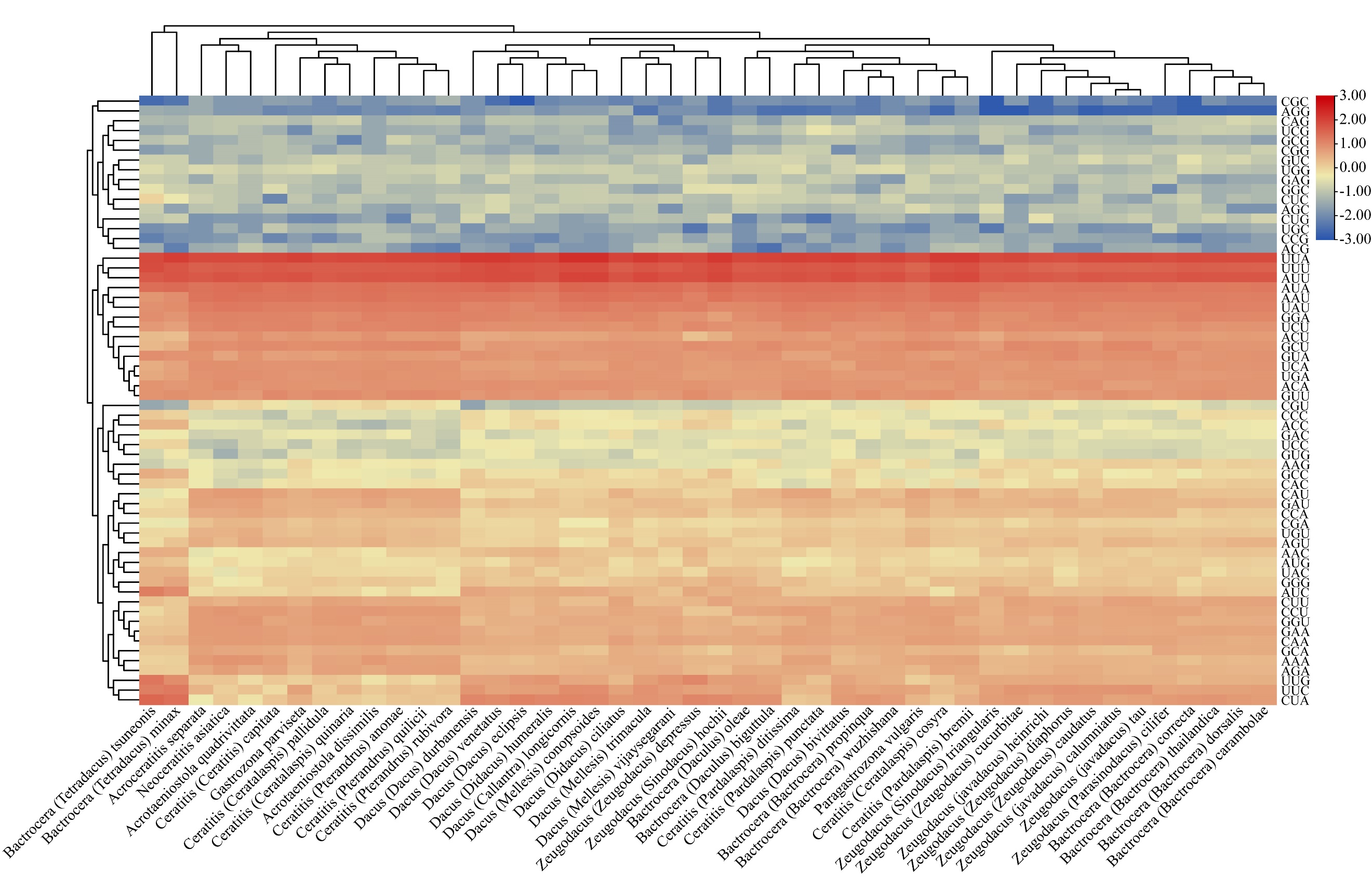


**Figure S2.** Hierarchical clustering analysis for the heatmap showing total codon counts of 46 mitogenomes species within Dacinae.


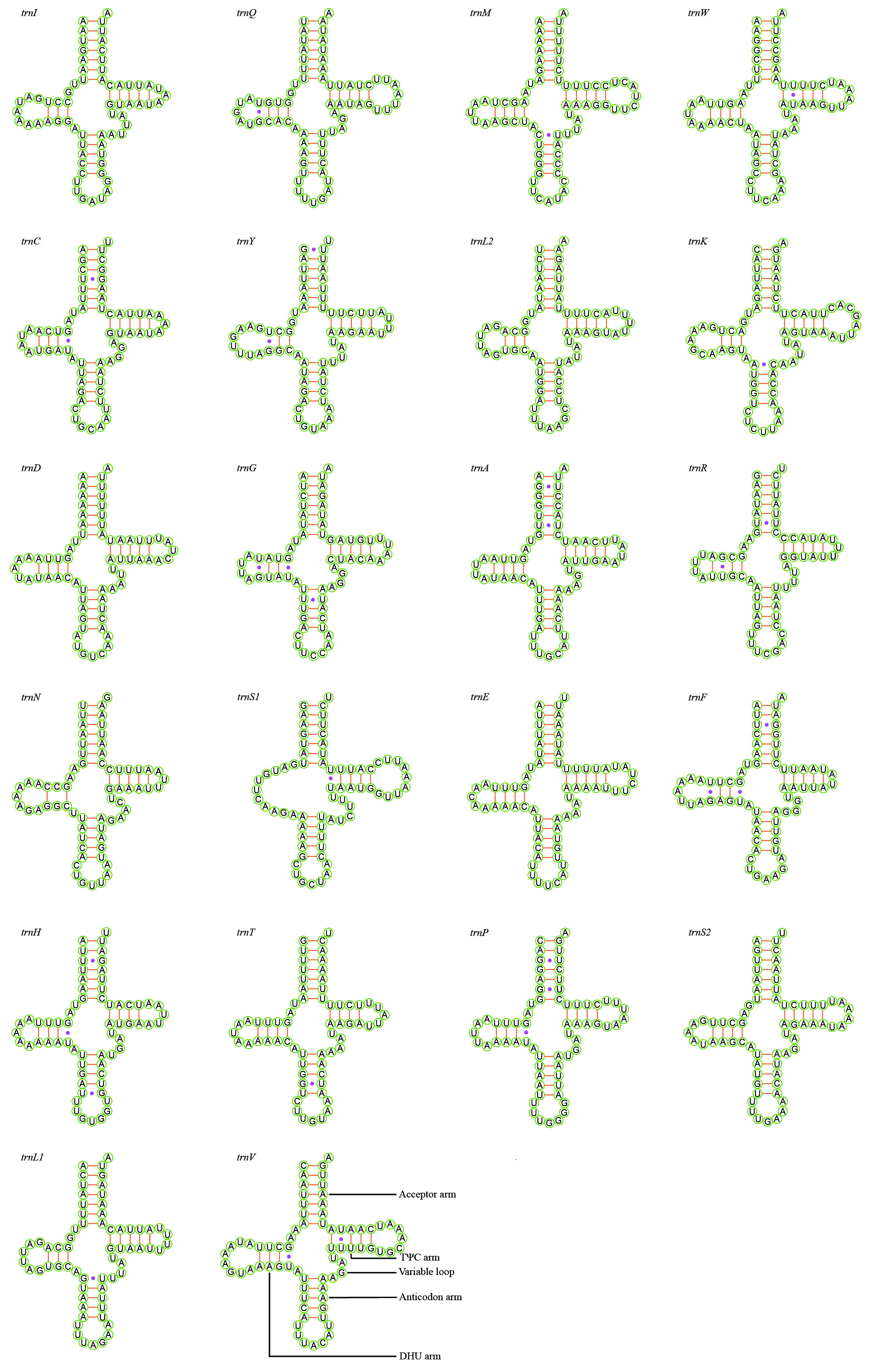
**Figure S3.** Predicted cloverleaf secondary structure for the 22 tRNAs of *Acroceratitis separata*.


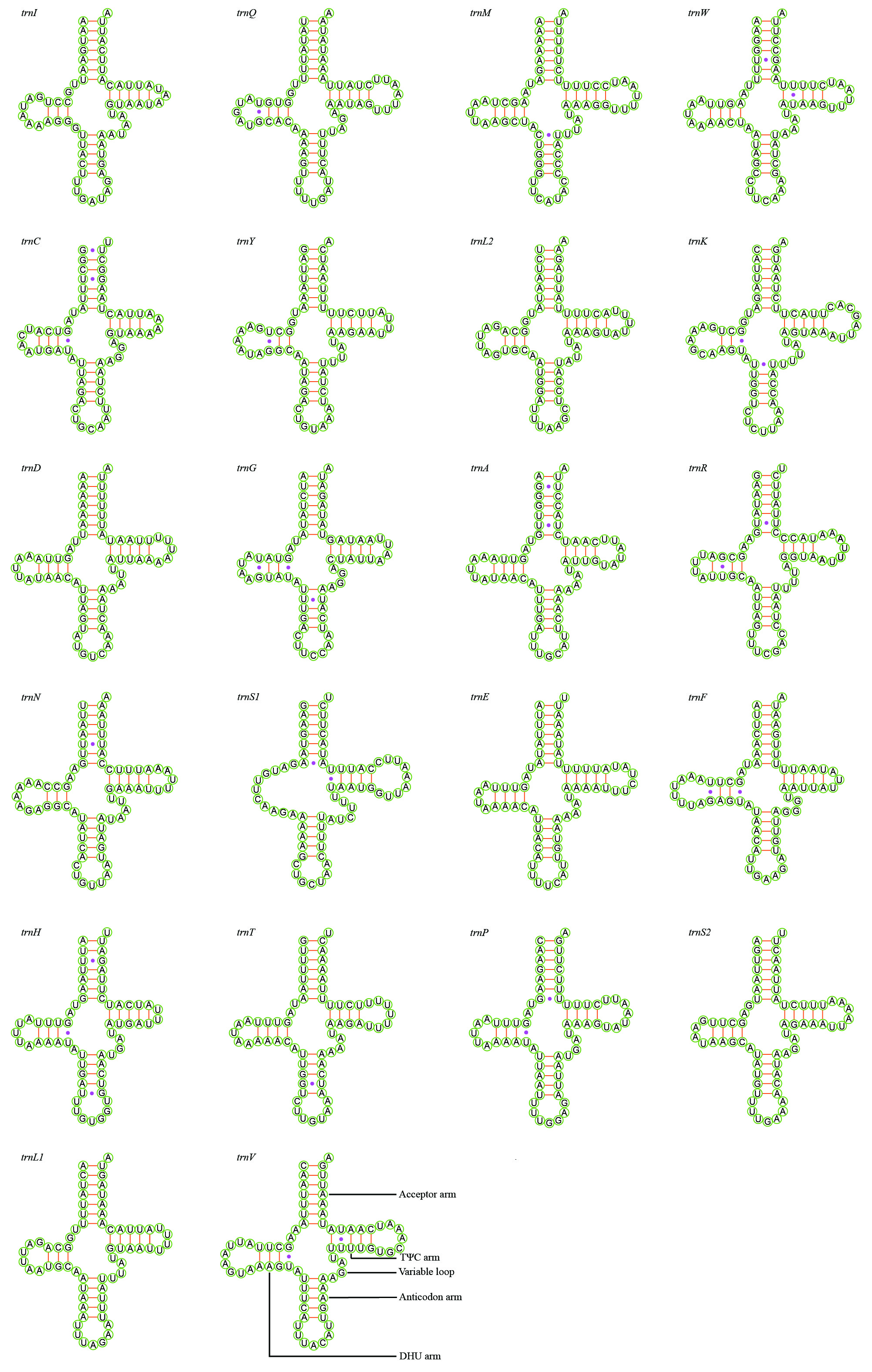
**Figure S4.** Predicted cloverleaf secondary structure for the 22 tRNAs of *Acrotaeniostola quadrivittata*.


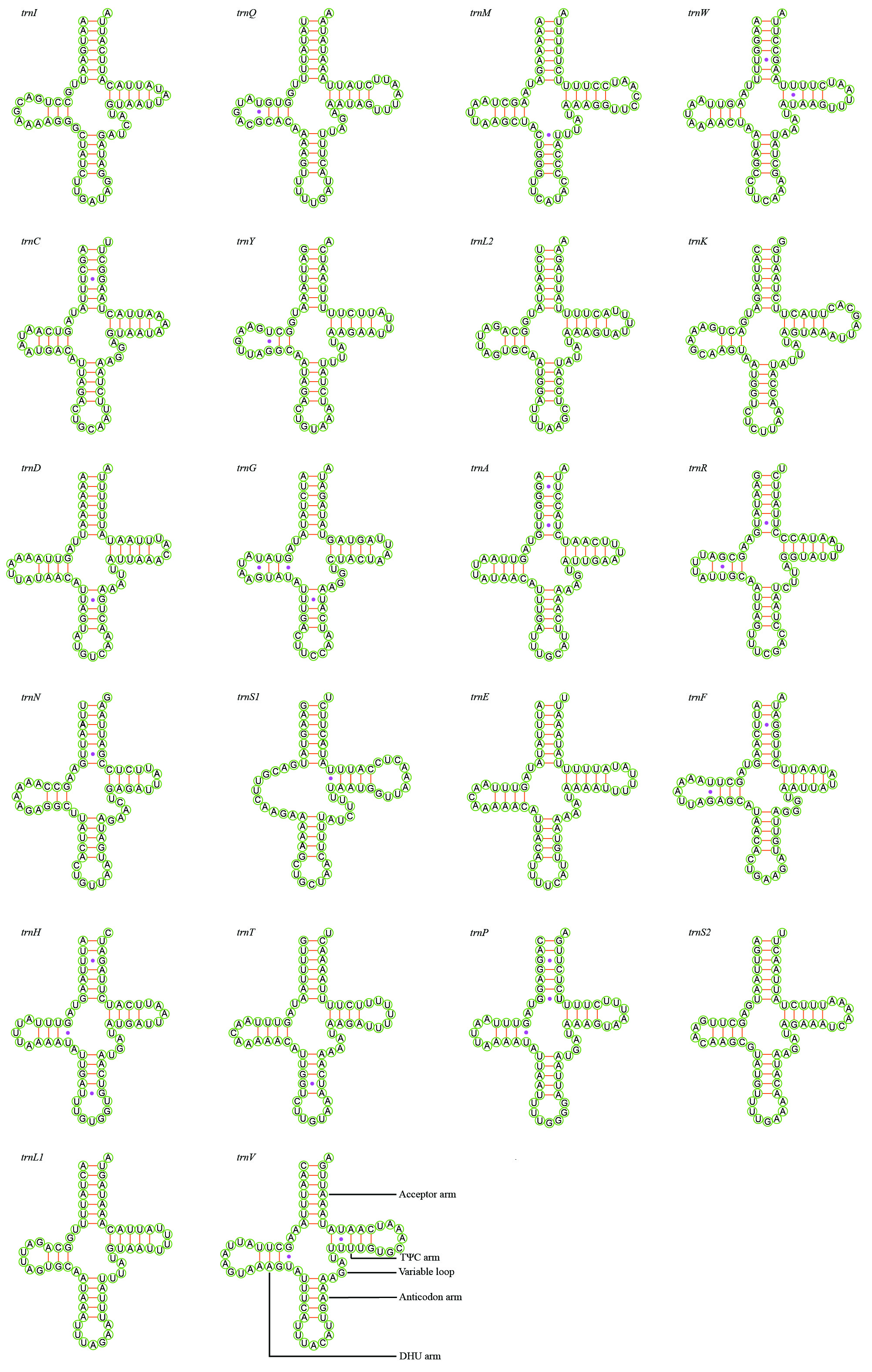
**Figure S5.** Predicted cloverleaf secondary structure for the 22 tRNAs of *Gastrozona parviseta*.


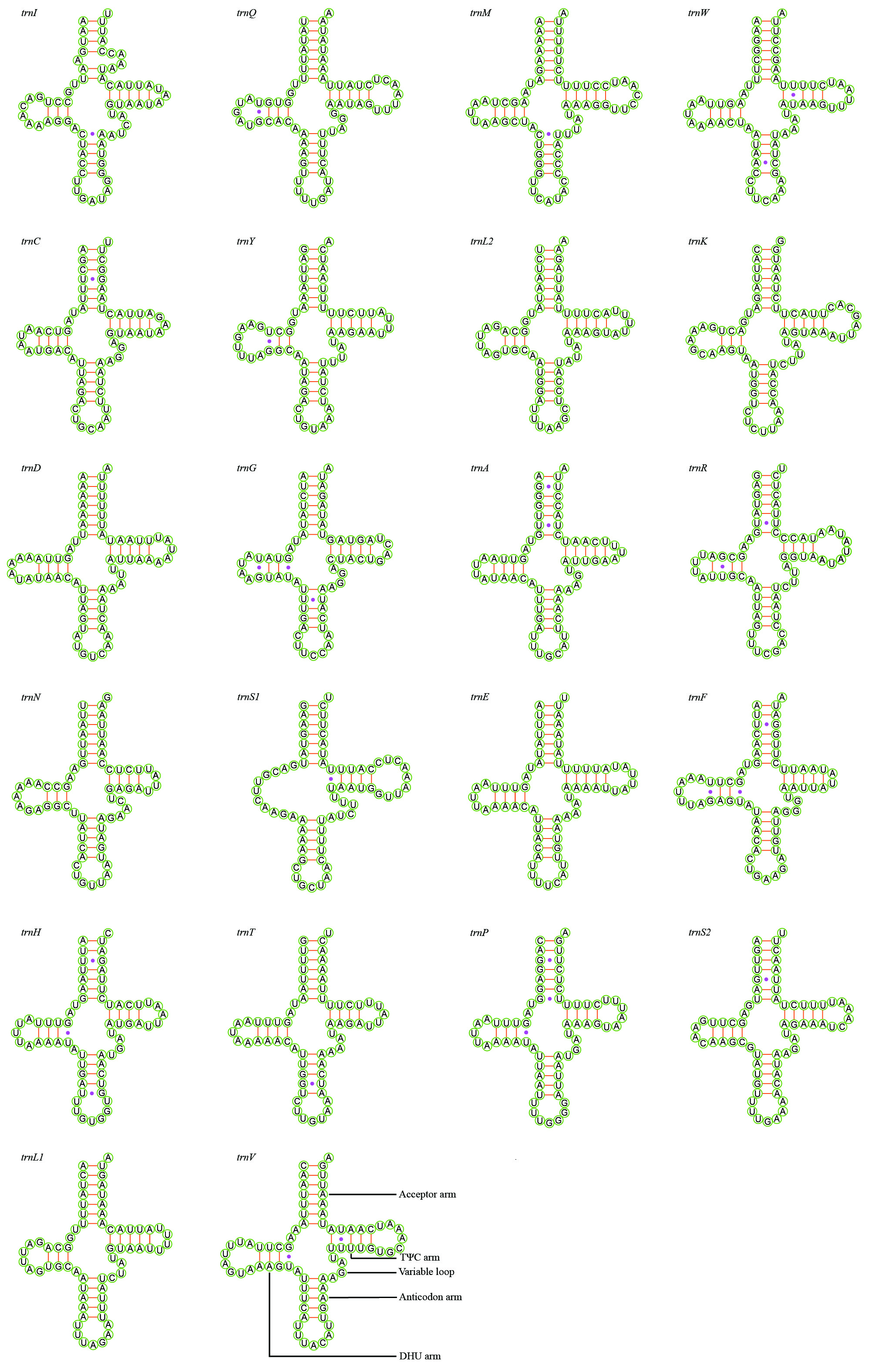
**Figure S6.** Predicted cloverleaf secondary structure for the 22 tRNAs of *Paragastrozona vulgaris*.


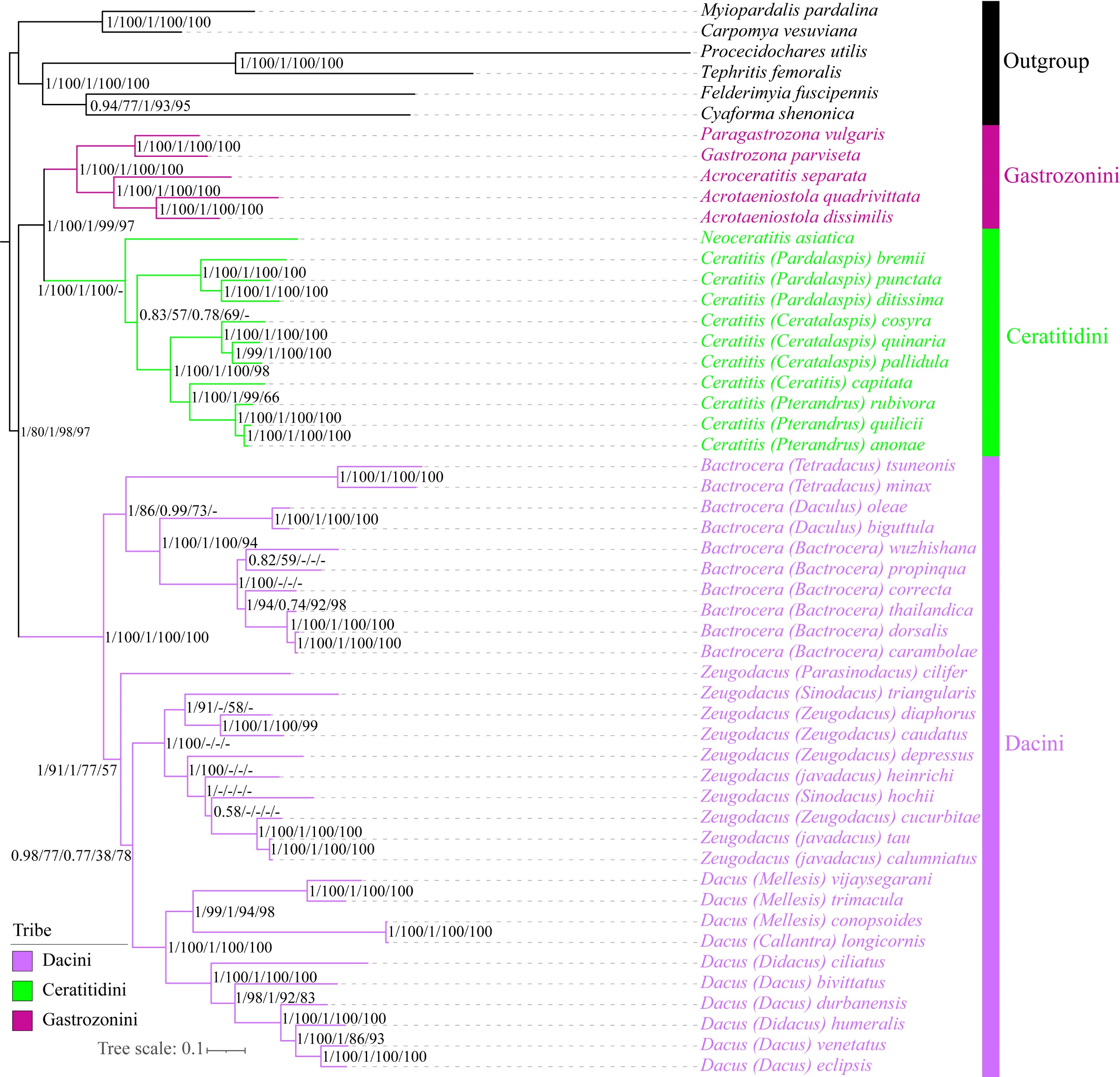


**Figure S7.** Phylogenetic tree of Dacinae inferred from the PCG123-BI + ML, PCG12R-BI + ML, and AA-ML datasets using Bayesian inference and Maximum likelihood methods. Numbers on branches are posterior probabilities and bootstrap support values.


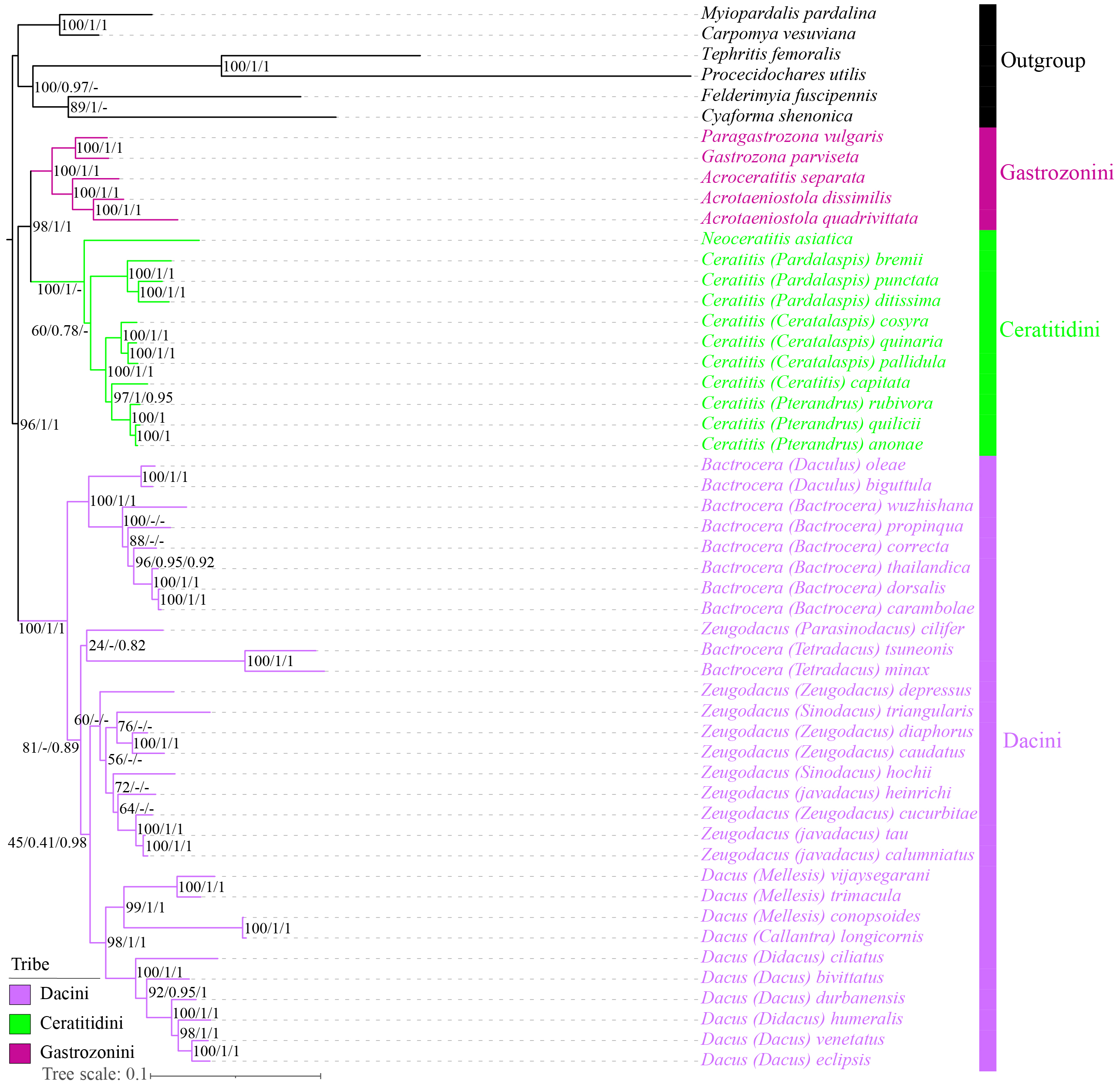


**Figure S8.** Phylogenetic tree of Dacinae inferred from the PCG12-ML + BI and AA-BI datasets using Maximum likelihood and Bayesian inference methods. Numbers on branches are bootstrap support and posterior probabilities values.


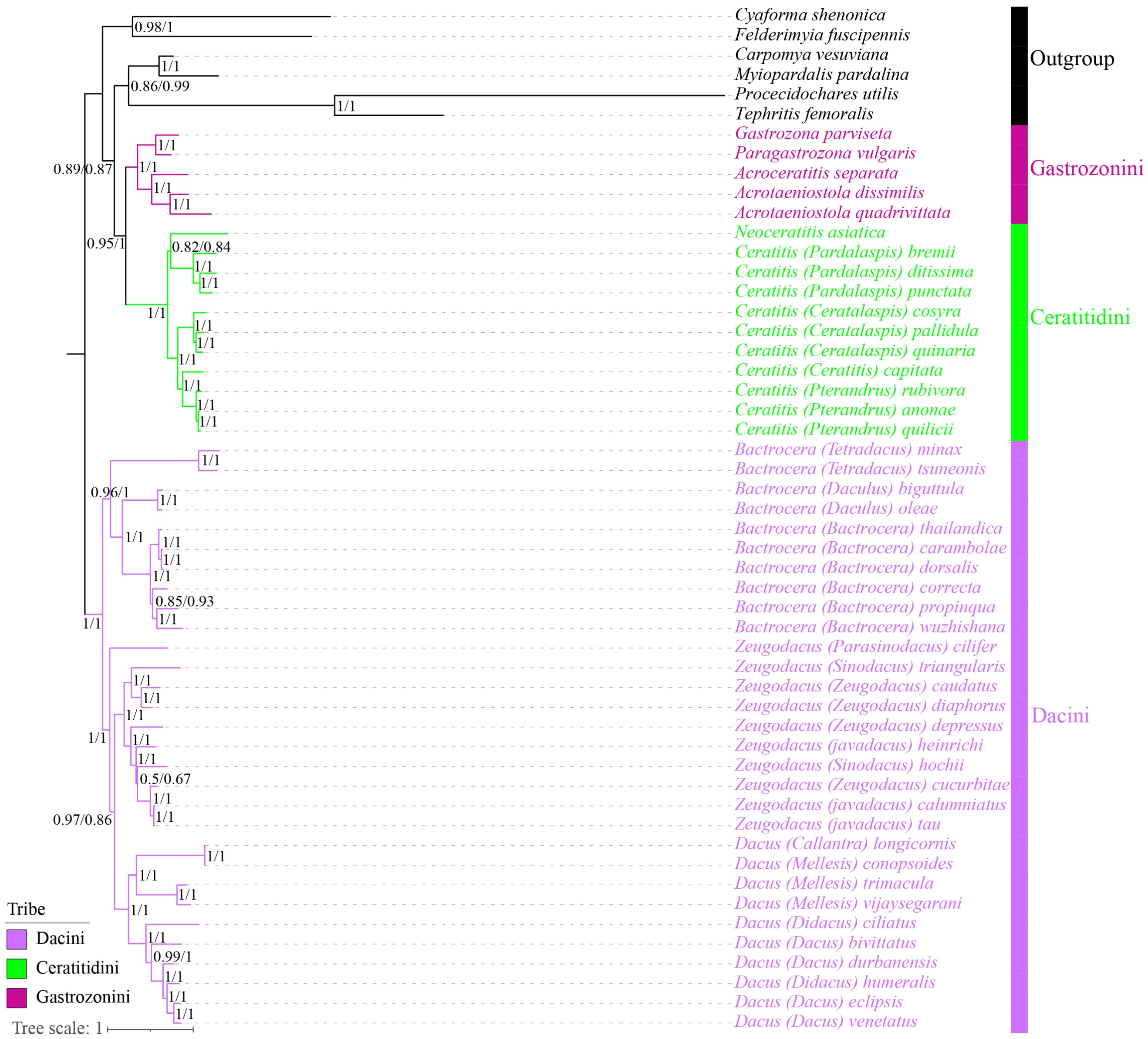


**Figure S9.** Phylogeny of Dacinae inferred from the PCG123 and PCG123R datasets using PhyloBayes analysis under the site-heterogeneous mixture model CAT + GTR. Supports at nodes are Bayesian posterior probabilities.


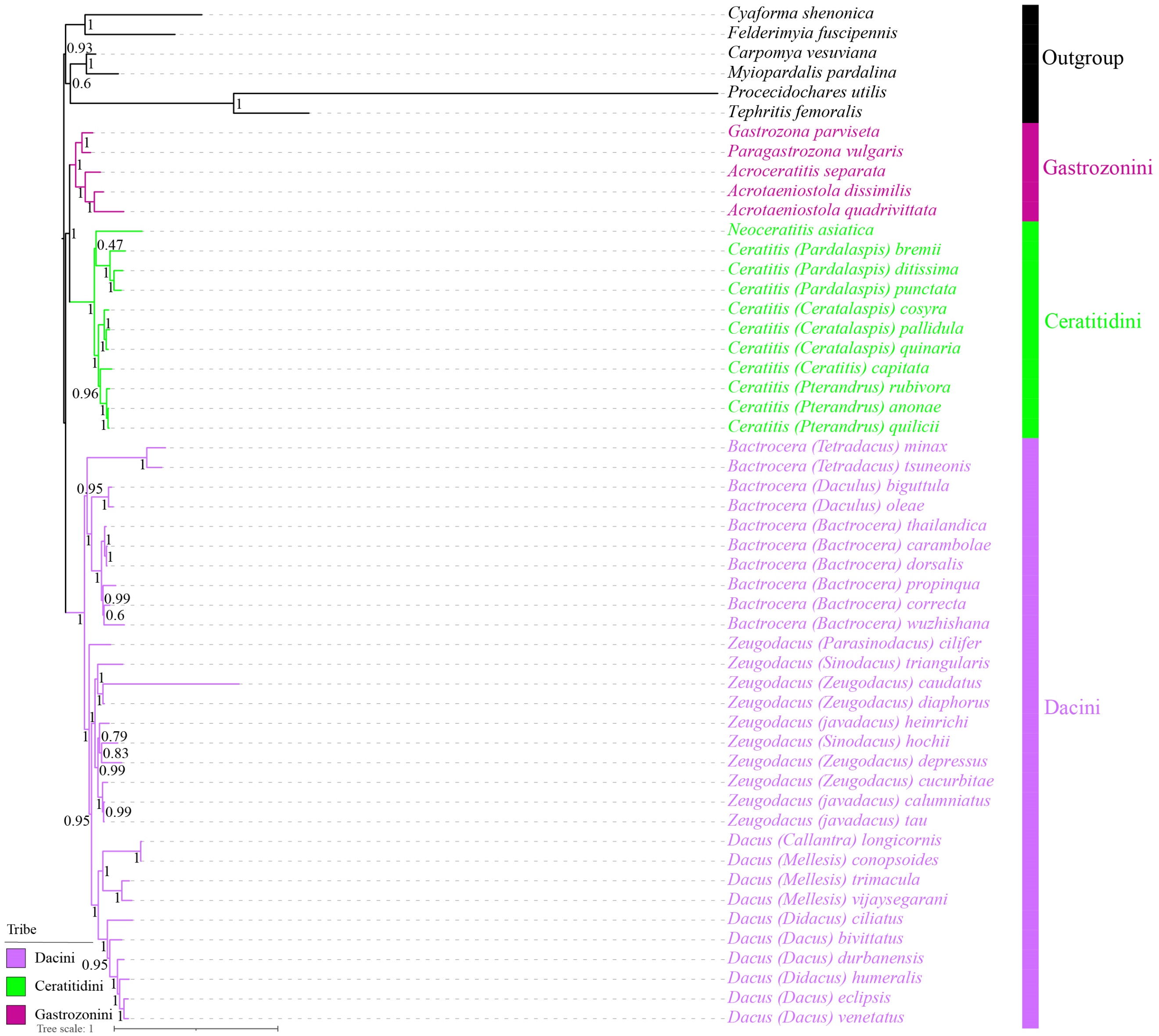


**Figure S10.** Phylogeny of Dacinae inferred from the PCG12R dataset using PhyloBayes analysis under the site-heterogeneous mixture model CAT + GTR. Supports at nodes are Bayesian posterior probabilities.

**
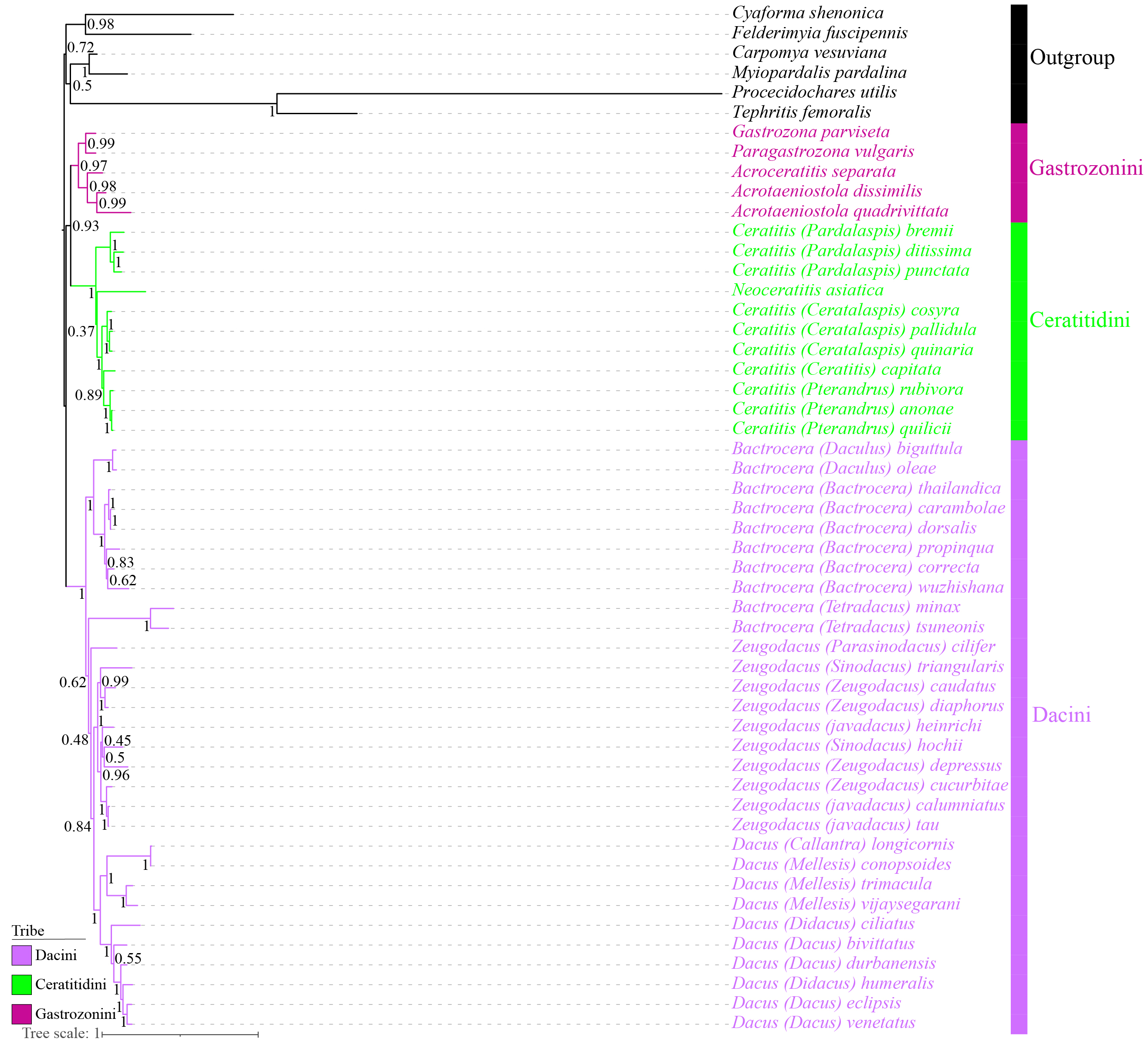
**

**Figure S11.** Phylogeny of Dacinae inferred from the PCG12 dataset using PhyloBayes analysis under the site-heterogeneous mixture model CAT + GTR. Supports at nodes are Bayesian posterior probabilities.
